# Impact of P‐wave indices in prediction of atrial fibrillation—Insight from loop recorder analysis

**DOI:** 10.1111/anec.12854

**Published:** 2021-05-07

**Authors:** Fabienne Kreimer, Assem Aweimer, Andreas Pflaumbaum, Andreas Mügge, Michael Gotzmann

**Affiliations:** ^1^ University Hospital St Josef Hospital, Cardiology and Rhythmology Ruhr University Bochum Germany; ^2^ University Hospital Bergmannsheil, Cardiology and Angiology Ruhr University Bochum Germany

**Keywords:** atrial fibrillation, implantable loop recorder, p‐wave indices

## Abstract

**Background:**

Several P‐wave indices are associated with the development of atrial fibrillation (AF). However, previous studies have been limited in their ability to reliably diagnose episodes of AF. Implantable loop recorders allow long‐term, continuous, and therefore more reliable detection of AF.

**Hypothesis:**

The aim of this study is to identify and evaluate ECG parameters for predicting AF by analyzing patients with loop recorders.

**Methods:**

This study included 366 patients (mean age 62 ± 16 years, mean LVEF 61 ± 6%, 175 women) without AF who underwent loop recorder implantation between 2010–2020. Patients were followed up on a 3 monthly outpatient interval.

**Results:**

During a follow‐up of 627 ± 409 days, 75 patients (20%) reached the primary study end point (first detection of AF). Independent predictors of AF were as follows: age ≥68 years (hazard risk [HR], 2.66; 95% confidence interval [CI], 1.668–4.235; *p* < .001), P‐wave amplitude in II <0.1 mV (HR, 2.11; 95% CI, 1.298–3.441; *p* = .003), P‐wave terminal force in V_1_ ≤ −4000 µV × ms (HR, 5.3; 95% CI, 3.249–8.636; *p* < .001, and advanced interatrial block (HR, 5.01; 95% CI, 2.638–9.528; *p* < .001). Our risk stratification model based on these independent predictors separated patients into 4 groups with high (70%), intermediate high (41%), intermediate low (18%), and low (4%) rates of AF.

**Conclusions:**

Our study indicated that P‐wave indices are suitable for predicting AF episodes. Furthermore, it is possible to stratify patients into risk groups for AF using simple ECG parameters, which is particularly important for patients with cryptogenic stroke.

## 1 INTRODUCTION

Atrial fibrillation (AF) is a common cardiovascular disease with significant impact on morbidity and mortality (Hindricks et al., 2020). Early detection of AF is particularly important because of its association with stroke. AF is a disease of the atria that is often associated with structural and functional changes. These changes in the atria are pre‐existing before the first onset of AF and can be partially detected on the ECG (Ciuffo et al., 2020). Since the P‐wave represents the electrical activation of the atria in the ECG, P‐wave indices in particular are suitable as predictors for the occurrence of AF (Aizawa et al., 2017; German et al., 2016; Rasmussen et al., 2020). In the past, it was possible to identify a number of ECG parameters that were associated with the later occurrence of AF (De Bacquer et al., 2007; Magnani et al., 2015). For example, in the past it was revealed that P‐wave duration >120 ms, P‐wave terminal force in V_1_, and P‐wave axis are associated with later AF (Aizawa et al., 2017; German et al., 2016; Magnani et al., 2015; Rasmussen et al., 2020). However, previous studies examining P‐wave indices have had limitations in detecting AF episodes. The diagnosis of AF was based on symptomatic episodes, incidental ECG documentation, or hospital admission for AF (Cheng et al., 2009; Eranti et al., 2020; Magnani et al., 2011; Nielsen et al., 2013, 2015; Soliman et al., 2009).

Recently, the Crystal AF study impressively demonstrated that loop recorder implantation in patients with cryptogenic stroke leads to a significantly higher rate of AF detection than conventional diagnostics. After 1 year, the diagnosis of AF was made more than 6 times more frequently in the group with loop recorder than in the control group (Sanna et al., 2014). It can therefore be assumed that loop recorders, due to their long‐term and uninterrupted monitoring, significantly improve the detection of AF. However, there are little data on predictors of AF detection in patients with loop recorders (Diederichsen et al., 2020; Melis et al., 2021). Furthermore, studies on the prognostic significance of P‐wave indices in patients with loop recorders are lacking.

The aim of the present study was therefore to investigate, for the first time, the association of frequent P‐wave indices with the occurrence of AF in a study cohort with implanted loop recorders. In addition, an attempt was made to develop an ECG‐based risk score that could divide patients into groups with low and high risk for the later occurrence of AF. Such an AF risk score would be particularly clinically important for patients with cryptogenic stroke.

## 2 METHODS

In this study, all patients who received an implantable loop recorder between 2010 and 2020 at the university hospitals of the Ruhr University Bochum St. Josef Hospital and Bergmannsheil Bochum were examined. Indications for the implantation of a loop recorder included syncope, cryptogenic stroke, and unclear palpitations. The loop recorders were manufactured by Medtronic (Reveal DX, Reveal XT, Reveal LINQ), St. Jude Medical (Confirm Rx), and Biotronik (BioMonitor, BioMonitor 2‐AF, Biomonitor III). Patients gave informed consent. All patients had a medical history, medication, laboratory results, ECG, and echocardiography prior to implantation.

This study is a retrospective analysis of prospectively obtained data. The study was approved by the local ethics committee of the Ruhr University Bochum (Number 21‐7155‐BR).

### 2.1 Inclusion and exclusion criteria, follow‐up, and endpoint

All patients with implanted loop recorders were examined in the corresponding ambulatories of the hospitals at intervals of 3 months. Additional outpatient visits were possible if the patients reported symptoms requiring clarification (e.g., syncope or palpitations). The outpatient follow‐up included the medical history and a query of the loop recorder.

Only patients with sinus rhythm were included in the analysis. Patients were excluded from the analysis if they had been diagnosed with AF in the past or if they had AF at the time of implantation. Patients who did not have any device interrogations reports after implantation were also excluded. The primary endpoint of the study was the first occurrence of AF.

The diagnosis of AF was established on the basis of the automatic detection of the device and after validation by a cardiologist. The minimum duration of AF detection in most implantable cardiac monitors is 2 min. In addition, we also interrogated each episode of arrhythmia recorded by the device and patient‐activated episodes. In the case where an episode of ≥30 s of irregular heart rhythm, without detectable P‐waves was recorded, the diagnosis of AF was made (Sanna et al., 2014). Follow‐up ended at the latest loop recorder check: either because of battery depletion, explantation of the system, or when the patients no longer attended outpatient follow‐up.

### 2.2 ECG analysis

All patients underwent a complete analysis of the 12‐lead ECG recorded within 24 h prior to implantation of the loop recorder. The standard 12‐lead surface ECG was recorded at a rate of 50 mm/s and a voltage of 10 mm/mV. All evaluations were conducted by a single observer who was blinded to the patients' group. The ECG analysis included in particular P‐wave indices but also an analysis of the QRS complex.

The P‐wave is an expression of atrial depolarization of first the right and then the left atrium. The maximum height of the P‐wave amplitude was determined in lead II. P‐wave duration was defined as the maximum P‐wave duration in one of the 12 leads. The P‐wave dispersion was calculated by subtracting the minimum duration of the P‐wave from the maximum duration of the P‐wave in the 12‐lead ECG (Dilaveris & Gialafos, 2001). The P‐wave axis was determined, with the range between 0° and 75° defined as normal (German et al., 2016). The range <0° was defined as left deviation and the range >75° as right deviation.

**FIGURE 1:**
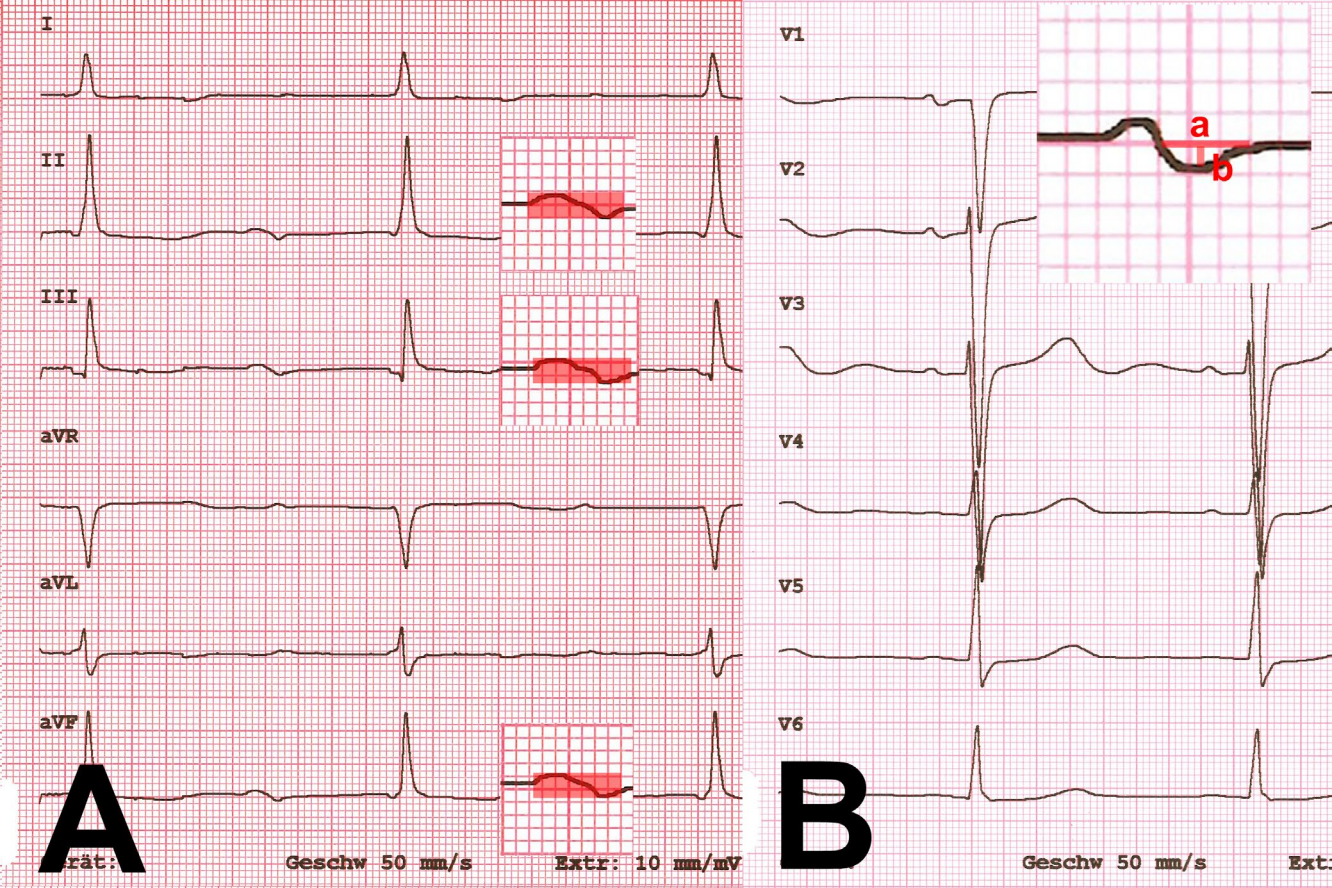
(A) Advanced interatrial block: prolongation of the P‐wave ≥120 ms in combination with a biphasic morphology of the P‐wave in lead II, III, and aVF, (B) the P‐wave terminal force in lead V_1_ (PTFV_1_) was calculated by a) width of the second term of the P‐wave multiplying b) the depth of this term of the P‐wave

An advanced interatrial block is a block of the interatrial conduction block in the Bachmann bundle and results in retrograde excitation of the left atrium. An advanced interatrial block was defined as a prolongation of the P‐wave ≥120 ms in combination with a biphasic morphology of the P‐wave in lead III and aVF, and a biphasic morphology or notched morphology of the P‐wave in lead II (German et al., 2016). The P‐wave in lead V_1_ is usually biphasic, with the second, negative term of the P‐wave representing the left atrial electrical activation. The P‐wave terminal force in lead V_1_ (PTFV_1_) was calculated by multiplying the depth of the second term of the P‐wave by the width of this term of the P‐wave (Figure 1) (German et al., 2016). The duration of the QRS complex was measured in the lead with the widest QRS complex. The axis of the QRS complex and the T wave were determined. Right and left bundle branch block were defined according to the usual criteria.

### 2.3 Statistics

Numerical values are expressed as mean ± standard deviation. Continuous variables were compared between groups using an unpaired *t* test (for normally distributed variables) or Mann–Whitney *U* test (for non‐normally distributed variables). Chi‐square analysis was used to compare categorical variables. All variables in Tables 1 and 2 were evaluated for the primary study end point in a univariate Cox proportional hazard model. All variables with a significant association were entered in a multivariate Cox model to identify independent predictors of outcome. Receiver operating characteristic curves were generated to define cutoff values for independent predictors. Freedom from AF was analyzed by the Kaplan–Meier method, and curves were compared by the log‐rank test. Independent predictors identified by the multivariate Cox proportional hazard survival model were used to derive a prognostic index to classify patients into different risk groups. Results are present as hazard risk. A *p* value <.05 was considered significant. All probability values reported are 2‐sided.

**TABLE 1:** Clinical characteristics of study patients (*n* = 366)

	Detection of AF (*n* = 75)	No detection of AF (*n* = 291)	*p* Value
Age (years)	69 ± 13	60 ± 16	<.001
Women (♀), *n* (%)	37 (49)	138 (47)	.764
Body mass index (kg/m^2^)	28 ± 5	28 ± 6	.912
Left ventricular ejection fraction (%)	61 ± 4	61 ± 6	.566
Left atrial diameter (mm)	38 ± 6	38 ± 5	.998
Medical history			
Arterial hypertension, *n* (%)	60 (80)	196 (67)	.033
Diabetes mellitus, *n* (%)	17 (23)	45 (15)	.138
Coronary artery disease, *n* (%)	12 (16)	42 (14)	.733
Previous stroke, *n* (%)	33 (44)	96 (33)	.075
Labor			
Creatinine (mg/dl)	0.9 ± 0.2	0.9 ± 0.3	.720
TSH (mIU/L) (Quartile)	1.3 (0.7–1.9)	1.4 (0.9–2)	.598
Medication			
Beta‐blocker, *n* (%)	27 (36)	87 (30)	.283
ACE‐inhibitors & ARB, *n* (%)	41 (55)	152 (52)	.649

Abbreviations: AF, atrial fibrillation; ARB, Angiotensin II receptor blockers; TSH, Thyroid‐stimulating hormone.

**TABLE 2:** ECG indices

	Detection of AF (*n* = 75)	No detection of AF (*n* = 291)	*p* Value
Heart rate (beats/min)	71 ± 13	71 ± 13	.949
P‐wave amplitude in II (mV)	0.11 ± 0.05	0.12 ± 0.05	.006
P‐wave duration (ms)	112 ± 24	105 ± 16	.001
P‐wave dispersion (ms)	21 ± 12	22 ± 11	.761
P‐wave axis (°)	51 ± 30	50 ± 24	.760
P‐wave right axis deviation, *n* (%)	7 (9)	14 (5)	.151
P‐wave left axis deviation, *n* (%)	3 (4)	6 (2)	.357
P‐wave terminal force in V_1_ (µV × ms)	−4653 ± 2183	−3276 ± 1983	<.001
Advanced interatrial block, *n* (%)	11 (15)	4 (1)	<.001
PR interval (ms)	191 ± 40	177 ± 33	.001
QRS duration (ms)	96 ± 19	95 ± 19	.780
QRS axis (°)	15 ± 39	18 ± 41	.524
Right bundle branch block, *n* (%)	4 (5)	22 (8)	.503
Left bundle branch block, *n* (%)	3 (4)	13 (4)	.860
T‐wave axis (°)	48 ± 42	43 ± 28	.308
QT interval (ms)	399 ± 30	394 ± 33	.425

Abbreviation: AF, atrial fibrillation.

## 3 RESULTS

Between 2010 and 2020, a total of 437 patients received a loop recorder. Of these patients, 63 had previously been diagnosed with AF or had AF at the time of loop recorder implantation. These patients were excluded from the analysis. Of the remaining 374 patients, 8 patients (2%) had to be excluded because there were no device interrogations reports. The remaining 366 patients formed the final study cohort. The mean age of the study cohort at implantation was 62 ± 16 years (minimum 18 years, maximum 92 years) and 175 of the patients were women (48%). Arterial hypertension was present in 256 patients (70%), diabetes mellitus in 62 patients (17%), and coronary artery disease in 54 patients (15%). The mean left ventricular ejection fraction was 61 ± 6%.

The indications for loop recorder implantation were syncope (*n* = 210, 57%), cryptogenic stroke (*n* = 101, 28%), unexplained palpitations (*n* = 38, 10%), and other indications (*n* = 17, 5%). Patients received the following devices: Medtronic (Reveal DX [*n* = 51], Reveal XT [*n* = 94], Reveal LINQ [*n* = 1166]), St. Jude Medical (Confirm Rx [*n* = 32]), and Biotronik (BioMonitor [*n* = 4], BioMonitor 2‐AF [*n* = 7], Biomonitor III [*n* = 12]).

### 3.1 Follow‐up and patient characteristics

The mean follow‐up time was 627 ± 409 days. Seventy‐five patients (20%) were diagnosed with AF during the observation period based on loop recorder analysis. The diagnosis of AF was made after a mean of 277 ± 238 days. Patients in whom AF could be detected were older compared to patients without AF (69 ± 13 years vs. 60 ± 16 years, *p* < .001) and had more often arterial hypertension (80% vs. 67%, *p* = .033). All other characteristics, especially left ventricular ejection fraction and left atrial diameter, showed no significant differences between the patient groups (Table 1).

### 3.2 ECG analysis and predictors of atrial fibrillation

Several p‐wave indices showed significant differences between patients with and without AF. In addition, a prolonged PR interval was associated with the occurrence of AF. ECG parameters of ventricular depolarization and repolarization showed no association with the occurrence of AF (Table 2). On univariate Cox analysis, age, arterial hypertension, P‐wave amplitude in II, P‐wave duration, PTFV_1_, advanced interatrial block, and PR interval were significantly related to the primary study end point (Table 3).

**TABLE 3:** Univariate analysis

	Hazard ratio	Confidence interval	*p* Value
Age ≥68 years	2.658	1.668–4.235	<.001
Arterial hypertension	1.849	1.050–3.257	.033
P‐wave amplitude in II <0.1 mV	2.113	1.298–3.441	.003
P‐wave duration ≥120 ms	2.437	1.546–3.839	<.001
PTFV_1_ ≤ −4000 µV × ms	5.297	3.249–8.636	<.001
Advanced interatrial block	5.014	2.638–9.528	<.001
PR interval ≥200 ms	1.986	1.249–3.156	.004

Abbreviation: PTFV_1_, P‐wave terminal force in V_1_.

Using receiver operating characteristic analysis, cutoff values for separating study patients were age ≥68 years (area under the curve [AUC] 0.651, CI 0.585–0.716, *p* < .001), P‐wave amplitude in II <0.1 mV (AUC 0.616, CI 0.542–0.689, *p* = .002), P‐wave duration ≥120 ms (AUC 0.577, CI 0.494–0.660, *p* = .037), PTFV_1_ ≤ −4000 µV × ms (AUC 0.727, CI 0.660–0.795, *p* < .001), and PR interval ≥200 ms (AUC 0.590, CI 0.515–0.665, *p* = .038). Stepwise multivariable analysis identified Age ≥68 years, P‐wave amplitude in II <0.1 mV, PTFV_1_ ≤ −4000 µV × ms, and advanced interatrial block as independent predictors of AF (Table 4).

**TABLE 4:** Multivariate analysis

	Hazard ratio	Confidence interval	*p* Value
Age ≥68 years	2.658	1.668–4.235	<.001
P‐wave amplitude in II <0.1 mV	2.113	1.298–3.441	.003
PTFV_1_ ≤−4000 µV × ms	5.297	3.249–8.636	<.001
Advanced interatrial block	5.014	2.638–9.528	<.001

Abbreviation: PTFV_1_, P‐wave terminal force in V_1_.

### 3.3 Atrial fibrillation risk score

**FIGURE 2:**
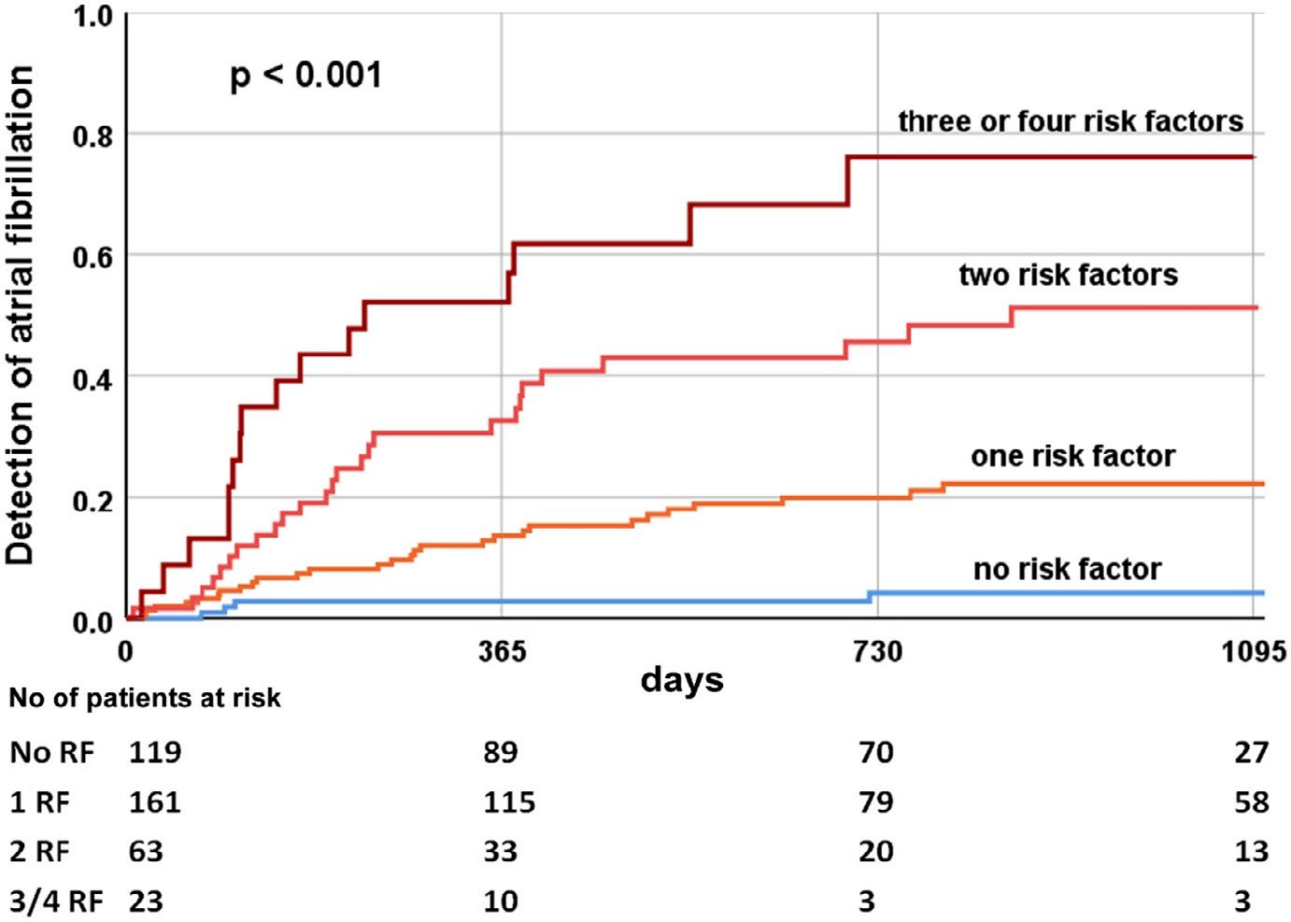
Kaplan–Meier estimates of atrial fibrillation in study patients (*n* = 366). Risk model based on independent predictors (age ≥68 years, P‐wave amplitude in II <0.1 mV, PTFV_1_ ≤ −4000 µV × ms, and advanced interatrial block)

Our predictive model was based on these four independent predictors: age ≥68 years, P‐wave amplitude in II <0.1 mV, PTFV_1_ ≤−4000 µV × ms, and advanced interatrial block. In patients without these risk factors, AF seldom occurred (<4%). The risk of AF increases with the number of independent risk factors (one risk factor, 18%; two risk factors, 41%; three and four risk factors, 70%; *p* < .001) (Figure 2).

### 3.4 Predictors of atrial fibrillation in patients with cryptogenic stroke

**FIGURE 3:**
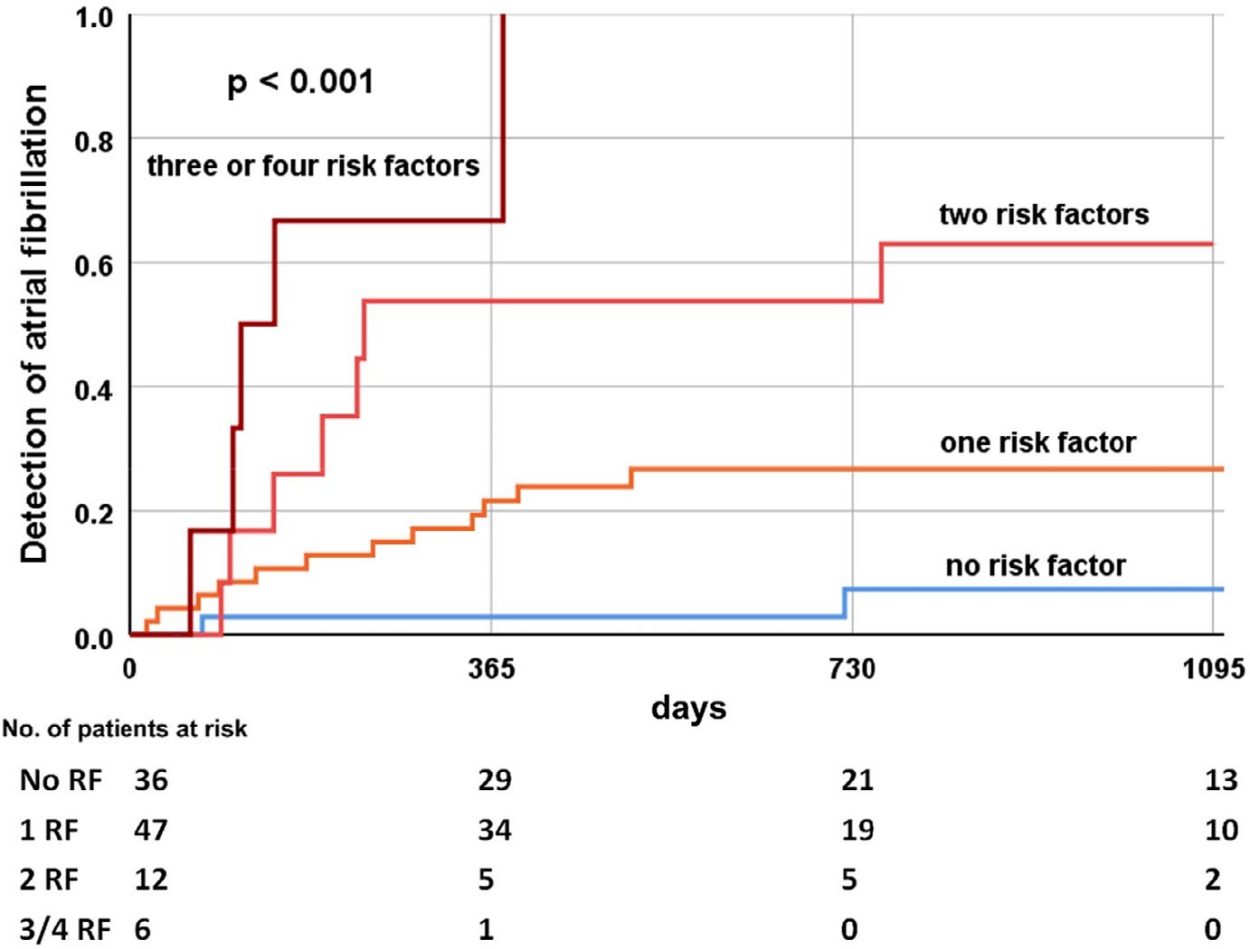
Kaplan–Meier estimates of atrial fibrillation in subgroup of patients with cryptogenic stroke (*n* = 101). Risk model based on independent predictors (age ≥68 years, P‐wave amplitude in II <0.1 mV, PTFV_1_ ≤ −4000 µV × ms, and advanced interatrial block)

Of the 366 patients in the study, 101 had a cryptogenic stroke as an indication for implantation of the loop recorder. In this subgroup, 26 patients (26%) had a new diagnosis of AF at follow‐up. We applied an AF risk score to this subgroup of patients and generated Kaplan–Meier curves. Even in patients with cryptogenic stroke, the risk score was able to differentiate patients at high and low risk for AF (5%, 25%, 59%, 83% *p* < .001) (Figure 3).

## 4 DISCUSSION

The present study investigated ECG parameters and clinical factors for the prediction of AF. For the first time, patients with implanted loop recorder were examined, in which the diagnosis of AF is more reliable than previously possible. The main findings of our study are that a number of P‐wave indices are predictors for the occurrence of AF in the next 9 months (on average AF occurred after 277 ± 238 days). The combination of the independent risk factors (age ≥68 years, P‐wave amplitude in II <0.1 mV, PTFV_1_ ≤ −4000 µV × ms, and advanced interatrial block) in a risk score is suitable to identify patients with low and very high risk for the first occurrence of AF in this period. This risk score is also suitable for estimating the probability of first occurrence of AF in patients with cryptogenic stroke.

### 4.1 P‐wave indices and atrial fibrillation

The P‐wave is generated by the electrical activation of the atria. It depends on several factors, especially on the size and hypertrophy of the atria but also on inter‐ and intra‐atrial conduction delays (Andlauer et al., 2018; Ciuffo et al., 2020; German et al., 2016; Josephson et al., 1977). Notably, in our study, the diameter of the left atrium as determined by echocardiography was not associated with the later occurrence of AF (Table 1). In contrast, several P‐wave indices were associated with the later onset of AF (Table 2). This observation suggests either that the P‐wave electrical alterations occur before left atrial enlargement or that the changes in P‐wave indices are an expression of delayed interatrial conduction. The latter assumption is supported by long known studies (Josephson et al., 1977).

In our study, P‐wave amplitude, P‐wave duration, PTFV_1_, advanced interatrial block, and PR interval were associated with AF (Table 2). Large epidemiological studies have already identified these P‐wave indices as risk factors for AF (Cheng et al., 2009; Eranti et al., 2020; Magnani et al., 2011; Nielsen et al., 2013, 2015; Soliman et al., 2009). As illustrated in the Crystal AF study, only a relatively small proportion of AF can be detected by conventional methods (Sanna et al., 2014). Therefore, it can be assumed that the detection of AF in the large epidemiological studies was limited and that a larger number of probands had short, asymptomatic periods of AF. The strength of our study is the detection of AF by means of loop recorder analysis. This made it possible to detect even asymptomatic, short phases of AF. Our study approach may therefore provide a more accurate insight into the significance of the P‐wave indices.

In our study, PTFV_1_ ≤ −4000 µV × ms was independently associated with the occurrence of AF after a mean of 9 months. PTFV_1_ is the product of the width and depth of the second, negative portion of the P‐wave in lead V_1_. This portion of the P‐wave represents electrical activation in the left atrium. Recently, Tiffany Win et al. demonstrated in a cardiac magnetic resonance imaging study that PTFV_1_ was associated with a higher degree of fibrosis in the left ventricle and impaired left atrial function (Tiffany Win et al., 2015). Our study supports previous studies that have already identified PTFV_1_ as an independent risk factor for AF (Eranti et al., 2020).

Advanced interatrial block was also independently associated with AF in our study. Advanced interatrial block results from block of the Bachmann bundle and is known to be associated with the development of various atrial tachyarrhythmias (van Campenhout et al., 2013). Deftereos et al. recently demonstrated in an electrophysiological study that this intra‐atrial conduction delay is a strong predictor for the development of AF (Deftereos et al., 2014). Our study cohort consisted of patients with cardiovascular risk factors and a mean age of 62 ± 16 years. However, advanced interatrial block was present in less than 4% of cases at baseline. Therefore, it can be concluded that a limitation of this significant risk factor in our study was that advanced interatrial block was relatively rare.

### 4.2 Risk score

Previously developed risk scores for the development of AF (Alonso et al., 2013; Chamberlain et al., 2011; Schnabel et al., 2009) have had limitations in detecting AF because short, asymptomatic episodes of AF might elude conventional diagnosis (Sanna et al., 2014). In our loop recorder study, we were able to detect episodes of AF more reliably than in previous studies. Our risk score used the combination of the independent risk factors (age ≥68 years, P‐wave amplitude in II <0.1 mV, PTFV_1_ ≤ −4000 µV × ms, and advanced interatrial block) to predict AF (Table 4). This score consists of relatively few factors that can be derived from a simple 12‐lead ECG. However, the greatest advantage of the score may be that it appears possible to stratify patients into those at very low risk and those at very high risk of AF based on the factors (Figure 2).

### 4.3 P‐wave indices and ischemic stroke

P‐wave indices might indicate atrial cardiomyopathy. In addition to predicting AF episodes, previous studies have also found evidence that P‐wave indices are predictors of ischemic stroke (Acampa et al., 2019; He et al., 2017; Li et al., 2020) and the development of dementia (Gutierrez et al.,^2019^). These studies support the concept that P‐wave indices (especially PTFV_1_, P‐wave duration, and maximum P‐wave area) indicate an important pathophysiological link between atrial cardiomyopathy, AF, and stroke (He et al., 2017). Clinically, this relationship is particularly important in patients with cryptogenic stroke. In our study, the indication for implantation of a loop recorder was cryptogenic stroke in 101 patients (28%). In the follow‐up of the study, loop recorder analysis detected a newly diagnosed AF in 26 patients (26%). In our study, we applied our AF risk score to the subgroup of patients with cryptogenic stroke. Also, in this subgroup, the independent risk factors of our study seem to be suitable for risk stratification (Figure 3). Our study could also be helpful in the further diagnosis of patients with cryptogenic stroke. The use of loop recorders could be adapted to the individual risk.

### 4.4 Limitations

The present study only examined patients with an indication for implantation of a loop recorder. These patients have more cardiac and non‐cardiac comorbidities than healthy persons. In particular, the proportion of patients with cryptogenic stroke is relatively high. AF is therefore generally more common than in the general population, so the results cannot be generalized.

Detailed echocardiography and the determination of laboratory parameters (e.g., B‐type natriuretic peptide and troponin) might have improved the prediction. However, the focus of the study was on ECG parameters that are simple, inexpensive, and reproducible. The main limitation is the relatively small number of patients. However, our study is the largest study of implanted loop recorders in which P‐wave indices as predictors of AF have been identified.

## 5 CONCLUSION

The strengths of the present study are the analysis of known P‐wave indices and the weighting of the different factors. Our risk score allows risk stratification for the occurrence of AF using a simple 12‐lead ECG. This may be particularly important for patients with cryptogenic stroke.

The results of our study are the first step toward an individualized diagnosis of cryptogenic stroke and should be evaluated in a prospective study.

## CONFLICT OF INTEREST

None.

## AUTHOR CONTRIBUTIONS

M.G. planned and supervised the study. F.B. performed the data collection and follow‐up and analyzed the ECG. A.A. and A.P. supported the data collection and ECG analysis. M.G. and F.B. performed the calculations. A.M. assisted in the interpretation of the results and worked on the manuscript. All authors discussed the results and commented on the manuscript.

## ETHICS STATEMENT

The study was approved by the local ethics committee of the Ruhr University Bochum (Number 21‐7155‐BR).

## Data Availability

Data available on request from the author.
